# Dynamic Development of White Lupin Rootlets Along a Cluster Root

**DOI:** 10.3389/fpls.2021.738172

**Published:** 2021-09-07

**Authors:** Tamara Le Thanh, Bárbara Hufnagel, Alexandre Soriano, Fanchon Divol, Laurent Brottier, Célia Casset, Benjamin Péret, Patrick Doumas, Laurence Marquès

**Affiliations:** BPMP, Univ Montpellier, CNRS, INRAE, Institut Agro, Montpellier, France

**Keywords:** white lupin, rootlet, cluster root, determinate growth, mineral nutrition

## Abstract

White lupin produces cluster roots in response to phosphorus deficiency. Along the cluster root, numerous short rootlets successively appear, creating a spatial and temporal gradient of developmental stages that constitutes a powerful biological model to study the dynamics of the structural and functional evolution of these organs. The present study proposes a fine histochemical, transcriptomic and functional analysis of the rootlet development from its emergence to its final length. Between these two stages, the tissue structures of the rootlets were observed, the course of transcript expressions for the genes differentially expressed was monitored and some physiological events linked to Pi nutrition were followed. A switch between (i) a growing phase, in which a normal apical meristem is present and (ii) a specialized phase for nutrition, in which the rootlet is completely differentiated, was highlighted. In the final stage of its determinate growth, the rootlet is an organ with a very active metabolism, especially for the solubilization and absorption of several nutrients. This work discusses how the transition between a growing to a determinate state in response to nutritional stresses is found in other species and underlines the fundamental dilemma of roots between soil exploration and soil exploitation.

## Introduction

Plant roots are adaptive systems able to adjust their architecture and physiology to their fluctuating environment in order to achieve efficient uptake of water and mineral nutrients (Rogers and Benfey, 2015). Among the mineral nutrients, phosphorus holds a special place. Indeed, roots take up inorganic phosphate (Pi) from the soil solution, but Pi is poorly soluble and promptly forms complexes with abundant cations in the soil such as calcium or magnesium, and becomes unavailable for plant nutrition (Holford, 1997). As a result, plants often experience Pi deprivation and have developed strategies to structurally and functionally adapt their root system. The modification of the root architecture in response to Pi deficiency has been widely studied in the model plant *Arabidopsis thaliana* (Williamson et al., 2001; López-Bucio et al., 2002; Al-Ghazi et al., 2003; Nacry et al., 2005; Péret et al., 2014). One of the most important changes observed in the adaptation of the *A. thaliana* root system to Pi deficiency, is the arrest of primary root growth and the increase in the number and length of lateral roots (Bouain et al., 2016; Balzergue et al., 2017). This developmental plasticity is based on *de novo* organogenesis of root meristems from primary root differentiated tissues that initiate the new lateral root primordia (Trinh et al., 2018). These primordia evolve into meristems expressing the same key regulators that the embryonic primary root meristem. Among these main root architecture regulators, the transcription factors WUSCHEL-RELATED HOMEOBOX 5 (WOX5), REPRESSOR OF WUSCHEL1 (ROW1), SCARECROW (SCR), SHORTROOT (SHR), PLETHORAs (PLTs), NAC DOMAIN CONTAINING PROTEIN 9 (FEZ) and SOMBRERO (SBR) play a critical role to build a well-defined cellular organization with a central stem cell niche surrounding the organizing center, providing all the cell types of the root and the root cap (Malamy and Benfey, 1997; Willemsen et al., 2008; Zhang et al., 2015; Goh et al., 2016; Du and Scheres, 2017). *Arabidopsis thaliana* root physiology is also altered during Pi deprivation with an increase in the exudation of protons, organic acids and enzymes such as acid phosphatases (Pantigoso et al., 2020). All these changes are orchestrated by a complex molecular pathway, named PSR for “Pi Starvation Response” (Chiou and Lin, 2011; Puga et al., 2017). Homologous genes involved in the PSR pathway have been identified in many other species, including major crops, showing that this response to Pi deficiency is highly ubiquitous (Secco et al., 2013; Lyu et al., 2016). However, white lupin (*Lupinus albus* L.) is a pulse crop in which root response to Pi deficiency is spectacular. Indeed, it produces very specialized roots, called cluster roots or proteoid roots, in response to Pi limitation (Johnson et al., 1996; Watt and Evans, 1999; Neumann and Martinoia, 2002). A cluster root is a lateral root along which hundreds of very short rootlets develop, forming one to several very dense clusters. Not only do these numerous rootlets improve the exchange surface between plant and soil, but they also modify the rhizosphere zone to improve mineral nutrition. Indeed, they secrete large amounts of organic acids, phosphatases and protons, resulting in an “exudative burst” that improves overall Pi solubilization and acquisition (Neumann and Römheld, 1999; Massonneau et al., 2001). It should be noted that these responses to Pi deprivation are quite similar to those observed in *A. thaliana*, but with a much larger scale (López-Bucio et al., 2003). White lupin cluster roots are therefore structural and functional units combining developmental and physiological processes that are particularly effective in improving Pi nutrition (Watt and Evans, 1999; Lambers et al., 2006).

In this perspective, white lupin root constitutes a very interesting model to understand how a root system can be up-graded for nutrient-efficiency acquisition. We recently obtained a high-quality genome of white lupin and transcriptomic data that have contributed to the understanding of the molecular changes involved in structural and physiological responses leading to cluster root formation and function (Hufnagel et al., 2020). The numerous rootlets appearing along the cluster root seem to follow an initial developmental pattern similar to the lateral root one (Gallardo et al., 2019). In this study, we combine anatomical, transcriptomic and functional approaches to describe the evolution of white lupin rootlets from their emergence to their final growth arrest, in order to better understand the developmental steps of these specialized organs. The rapid and successive emergence of rootlets, along lateral roots, leads to the formation of a continuous spatial gradient of rootlets bearing all developmental stages. We took advantage of this patterning to use one cluster of rootlets as a very fine developmental model for studying the evolution of rootlet structure and function dedicated to soil exploration (foraging) and Pi absorption (mining), which are the two enter points to improve water and nutrient acquisition efficiency in plants.

## Materials and Methods

### Plant Materials and Growth Conditions

Seeds of white lupin (*L. albus* cultivar Amiga from Florimond Desprez, France), of 8 mm size, were used in all experiments. Seedlings were cultivated in growth chambers under controlled conditions (16 h light/8 h dark, 25°C day/20°C night, 65% relative humidity and PAR intensity 200 μmol m^–2^s^–1^). Seeds were germinated on vermiculite substrate for 4 days then either transplanted in 1.6 L pots or 200 L tanks depending on the experiments. The hydroponic solution was modified from Abdolzadeh et al. (2010) without phosphate: MgSO_4_ 54 μM; Ca(NO_3_)_2_ 400 μM; K_2_SO_4_ 200 μM; Na-Fe-EDTA 10 μM; H_3_BO_3_ 2.4 μM; MnSO_4_ 0.24 μM; ZnSO_4_ 0.1 μM; CuSO_4_ 0.018 μM and Na_2_MoO_4_ 0.03 μM. The solution was continuously aerated and was renewed every 7 days for pots.

### Molecular Cloning

We focused on three genes known to be involved in the network controlling the meristem maintenance in the model plant *A. thaliana: WUSCHEL-RELATED HOMEOBOX 5 (WOX5), SCARECROW (SCR)* and *REPRESSOR OF WUSCHEL 1 (ROW1)* (Drisch and Stahl, 2015). Taking advantage of our previously acquired genomic and transcriptomic data (Hufnagel et al., 2020), we selected white lupin genes with both high sequence homologies with *A. thaliana* genes and a high expression level in the cluster root: Lalb_Chr04g0250731 and Lalb_Chr18g0052701 for *LaWOX5.1-like* and *LaWOX5.2-like*, respectively, Lalb_Chr01g0019561 for *LaROW1-like* and Lalb_Chr19g0123861 for *LaSCR-like* (^1^ Supplementary Figure 1). Promoter sequences of *pLaWOX5.1-like* (Lalb_Chr04g0250731), *pLaWOX5.2-like* (Lalb_Chr18g0052701), *pLaROW1-like* (Lalb_Chr01g0019561) and *pLaSCR-like* (Lalb_Chr19g0123861) were extracted from white lupin genome [(Hufnagel et al., 2020) (see text footnote 1)]. *pLaSCR-like* was cloned according to Sbabou et al. (2010). The primers for *pLaWOX5-like* (F-5′- GGACGCTATAAAAGAATCACA-3′; R-5′- GCTCAATGATTC TGTGCCTCT-3′) and *pLaROW1-like* (F-5′- CGAGGAGCT TGAGTTGTCTCC -3′; R- 5′- ATCCATTGTCCATTTAGAAT TGC-3′) were designed using Primer3Plus (Untergasser et al., 2012). They were used to amplify, respectively, a total of 2,032 bp and 1,181 bp upstream of the start codon of *LaWOX5-like* and *LaROW1-like* from white lupin genomic DNA with the addition of the attb1 (5′-GGGGCCAAGTTTGTACA AAAAAGCAGGCT-3′) and attb2 (5′-CCCCCCACTTTGT ACAAGAAAGCTGGGT-3′) adapters. Amplified fragments were cloned into the pDONR221, then cloned into the binary plasmid pKGW-FS7 (Karimi et al., 2002) by Gateway reaction following the manufacturer’s instruction (Thermo Fisher). The pKGW-FS7 contains the glucuronidase gene (GUS) used as a reporter gene and the red fluorescent (DsRed) gene, under the control of an ubiquitine promoter, to control the transformation status of the hairy roots.

### Hairy Root Transformation of White Lupin

Hairy root transformation of white lupin was performed following protocol previously described (Gallardo et al., 2019), with the following modifications: six days after transformation, seedlings were transferred to vermiculite and put into little greenhouses to increase humidity level. Ten days later, 8 plants growing hairy roots were transferred into 1.6 L pot. After 8 to 12 days of culture in hydroponic conditions, roots were sampled and screened for red fluorescent protein (DsRed) as an *in vivo* marker for transformation allowing the selection of transformed roots. Each transformed root represents an independent transformation event.

### Histochemical Analysis

Histochemical staining of *β* -glucuronidase was performed on transformed hairy roots. Roots were incubated in a phosphate buffer containing 1 mg/mL X-Gluc as a substrate (X-Gluc 0.1%; phosphate buffer 50 mM, pH 7, potassium ferricyanide 2 mM, potassium ferrocyanide 2 mM, Triton X-100 0.05%) during 60 to 90 min and then washed before fixation.

### Fixation and Clearing Procedure

Roots dedicated to agarose inclusion were fixed with 4% paraformaldehyde (PFA) during 120 min at room temperature under vacuum treatment. The roots were then washed twice 2 min in 1X PBS and moved to the clearing solution ClearSee (Ursache et al., 2018) under vacuum treatment for 48 h. ClearSee solution was prepared by mixing 10% (w/v) Xylitol (W5079 Sigma), 15% (w/v) Sodium deoxycholate (D6750 Sigma) and 25% (w/v) Urea (EUOD14-D Euromedex) until complete dissolution of powders. Clearing solution was changed every day. Roots dedicated to resin inclusion were incubated in a fixative solution containing 2% formaldehyde, 1% glutaraldehyde and 1% caffeine for 2 h at 4°C.

### Microscope Analysis of Resin-Included Samples

For resin inclusion, roots were dehydrated in successive ethanol baths: 50% (30 min), 70% (30 min), 90% (1 h), 95% (1 h), 100% (1 h) and 100% (overnight). Samples were impregnated with half pure ethanol and half Technovit 7100 resin (Heraeus Kulzer, Wehrheim, Germany) for half a day, then in 100% resin for 2 days. Roots were then embedded in resin following the manufacturer’s recommendations. Thin resin sections of 8 μm were produced using a microtome (RM2165, Leica Microsystems, Wetzlar, Germany) then stained with 4,6-diamidino-2-phenylindole (DAPI) for 5 min in the dark. Staining solution was rinsed with MilliQ water. Resin sections were mounted in water and observed either with an Olympus BX61 epifluorescence microscope (Tokyo, Japan) or a Zeiss observer 7/ApoTome.

### Microscope Analysis of Agarose-Included Samples

For agarose inclusion, 14 days-old PFA-fixed and ClearSee-cleared cluster roots were transferred in 0.001‰ Calcofluor-ClearSee solution (Fluorescent Brightner 28 F3543 Sigma) during 1 h 30 under vacuum treatment. Roots were washed in 2 ClearSee baths for 10 min and then included in 4% (m/v) agarose. Thin sections of 80 to 100 μm were cut with a vibratome (Microcut H1200, Bio Rad, Hercules, CA), mounted in ClearSee and observed with confocal laser scanning microscopy (Leica SP8 microscope) with a 20X water objective and the 401 nm line. Fluorescence emission was collected from 420 to 500 nm. Images were analyzed with Fiji Software and cell lengths were measured with Fiji macro Cell-O-Tape (French et al., 2012), along 300 μm following a cortical cell line starting from the organization center.

### Transcriptome Analysis

RNA-seq raw data were obtained by the team (Hufnagel et al., 2020) and are available from the white lupin publicly shared database (see text footnote 1). Briefly, after 12 days of culture, ten cluster roots coming from four plants were harvested and dissected in seven sections from the apex. Four biological replicates were produced. Independent cluster root RNA-seq libraries were constructed and sequenced at Get-PlaGe core facility (INRAE, Toulouse, France). Raw reads have been cleaned using Cutadapt (Martin, 2011), and then mapped on the white lupin genome using Hisat2 (Kim et al., 2019). Gene count data has been obtained using StringTie (Pertea et al., 2015). In this study, we extended previous study by re-analyzing the RNA-seq data using DIANE web interface, a recent application for the analysis of high throughput gene expression data (Cassan et al., 2021) in order to provide information on the global gene activity during rootlet life. We selected the differentially expressed transcripts (DETs) between the section with pre-emerging rootlets (S1) and the section with rootlets that have just reached their final size (S7) using absolute log2(Fold Change) > 1 and False Discovery Rate (FDR) < 0.01. A clustering of the normalized gene expression profiles along cluster root of the 7,234 DETs obtained, has then been performed with the Coseq package in DIANE website, using Poisson mixture model (Cassan et al., 2021).

### Sampling and Expression Analysis of Cluster Root Genes (qRT-PCR)

Fourteen days after germination, six different types of 0.5 cm-long fragments were dissected along the cluster roots: (i) cluster root tip, (ii) stage II, (iii) stage III, (iv) stage IV, (v) stage V, and (vi) link. The link segment, corresponding to the attachment of the cluster root to the primary root, where no rootlets develop, was used as a control. Total RNA was extracted for each sample using the Direct-zol RNA MiniPrep kit (Zymo Research, Irvine, CA). RNA concentration was measured on a NanoDrop spectrophotometer (ND1000). cDNAs were prepared with 2 μg total RNA using the revertaid First Strand cDNA Synthesis (Thermo Fisher). Gene expression was quantified by quantitative Real Time -- Polymerase Chain Reaction (qRT-PCR) (LightCycler 480, Roche Diagnostics, Basel, Switzerland) using the SYBR Premix Ex Taq (Tli RNaseH, Takara, Clontech, Mountain View, CA). Specific primer pairs were designed with Primer3plus^2^ (Supplementary Table 2). Expression levels were normalized to a putative initiation factor eIF-4 gamma *LaNORM1* (Lalb_Chr07g0195211). Relative gene expression levels were calculated according to the ΔΔCt using the link sample as a calibrator. Four biological replicates were performed.

### Pi Influx Measurement in Rootlets

Cluster roots of 14 days old seedlings were dissected the same way as for the qRT-PCR analysis. Each sample contained 6 segments of 0.5 cm and triplicates were collected. Segments were washed 2 min in cold solution (MES 2 mM pH 4.5, CaSO_4_ 0.2 mM, NaH_2_PO_4_ 50 μM) and then incubated 45 min with 0.2 μCi ^33^P/mL. Finally, segments were washed 3 times 15 s in 0.2 mM CaSO_4_ kept on ice and transferred into 0.1 N HCl for 30 min under agitation. We aliquoted 300 μL of HCl solutions in 5 mL counter tubes already containing 3 mL of scintillation liquid. Radioactivity was counted with the Liquid Scintillation Counter TRI-CARB (Packard 2100TR). The link segment, where no rootlets were developed, was used as a control.

### Malate and Citrate Exudation in Rootlets

Cluster roots of 14 days old were dissected the same way as for the qRT-PCR analysis and five replicates were performed. For each developmental stage, the dissected fragments were put into Eppendorf tubes containing 1 mL of MilliQ water and agitated for 3h. The solution was used to quantify organic acids by ion exchange chromatography ICS-5000 HPIC system (Thermo Scientific Dionex), using an IonPac^®^ AS11 analytical column (250 mm × 4mm) with an isocratic gradient of 4 to 28 mM KOH for 16 min, with a volume of sample injected of 25 μL.

### Statistical Analysis

The results are presented as means ± standard error (SE). Statistical analysis was performed using GraphPad Prism 9. Data were analyzed by ordinary one-way analysis of variance (ANOVA) and means were compared using Tukey multiple comparisons test at *p* < 0.05 to determine significant differences.

## Results

### Characterization of Rootlets Development

**FIGURE 1:**
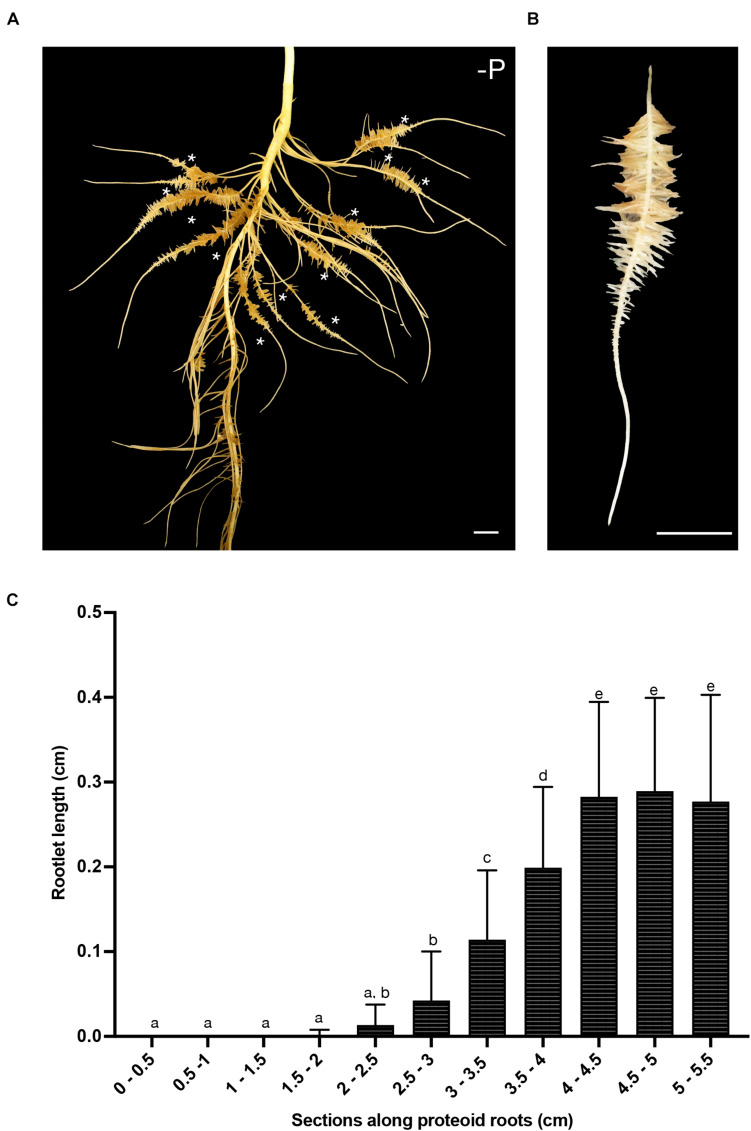
White lupin root system architecture. **(A)** Root system of a 16-days-old white lupin hydroponically grown in Pi deficiency conditions resulting in the development of several cluster roots each bearing a single cluster of rootlets (*). **(B)** A typical 7 cm-long cluster root displaying one cluster of rootlets. **(C)** Measurement of rootlet length: 5 rootlets were measured in 0.5 cm sections from the tip of the root to the end of the cluster (*n* = 21). Scale bars: **(A–B)** 1 cm.

The present study focused on the root systems of 16-days-old white lupin that produced several cluster roots in their upper part in response to phosphorus starvation (Figure 1A). Each young cluster root carried a single cluster of third-order lateral roots with a determinate growth, named rootlets (Figure 1B). In our growing conditions, these rootlets reach a final length of about 3 mm in two to three days (Figure 1C). These rootlets successively emerge along the cluster root, and within a cluster, the youngest ones are always at the tip-side of the cluster root.

**FIGURE 2:**
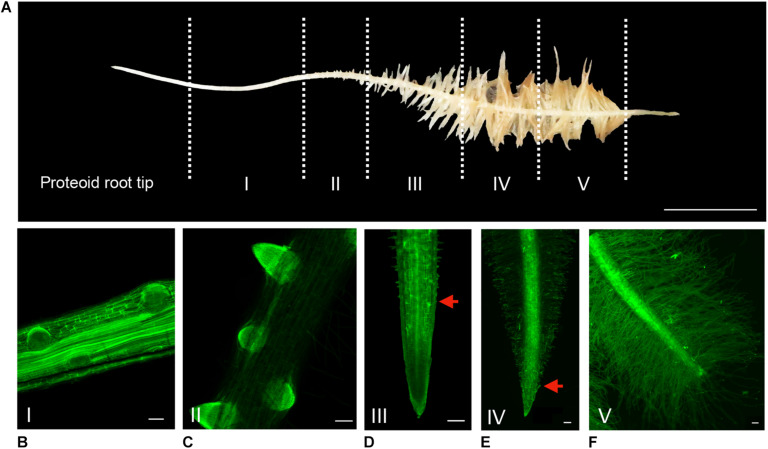
Spatio-temporal evolution of rootlet development. **(A)** Division of a cluster into 5 stages of rootlet development. Calcofluor was used to stain 100-μm thin longitudinal sections of rootlets at **(B)** Stage I: pre-emergence. **(C)** Stage II: emergence. **(D)** Stage III: growing. **(E)** Stage IV: rootlets having reached their maximal length. **(F)** Stage V: fully differentiated rootlets. Red arrows indicate the closest root hair from the tip. Scale bars: **(A)** 1 cm, **(B–F)** 100 μm.

Along a cluster, the spatial gradient representing the dynamic evolution over time of rootlet development has been divided into five typical stages (Figure 2A). To investigate the different morphological stages of rootlet development, the cluster roots were stained with the fluorescent calcofluor−white dye which labels cellulose in cell walls. We defined stage I as the pre-emerging state in which the rootlet primordia are formed but have not yet cross the last layer of the cluster root epidermis (Figure 2B). In stage II, the rootlet has completely emerged and comes into contact with the rhizosphere (Figure 2C). Then, during stage III, a growing phase occurs while root hairs appear in the differentiating zone (Figure 2D). In stage IV, rootlets reach their final length and root hairs start to overrun the tip (Figure 2E). Finally, in stage V root hairs cover the entire rootlet including the tip (Figure 2F). These events are continuous and can be followed spatially along one young cluster. The presence of root hairs around the tip at stage V suggests a complete differentiation of all the rootlet cells and hypotheses the disappearance of the meristematic zone.

**FIGURE 3:**
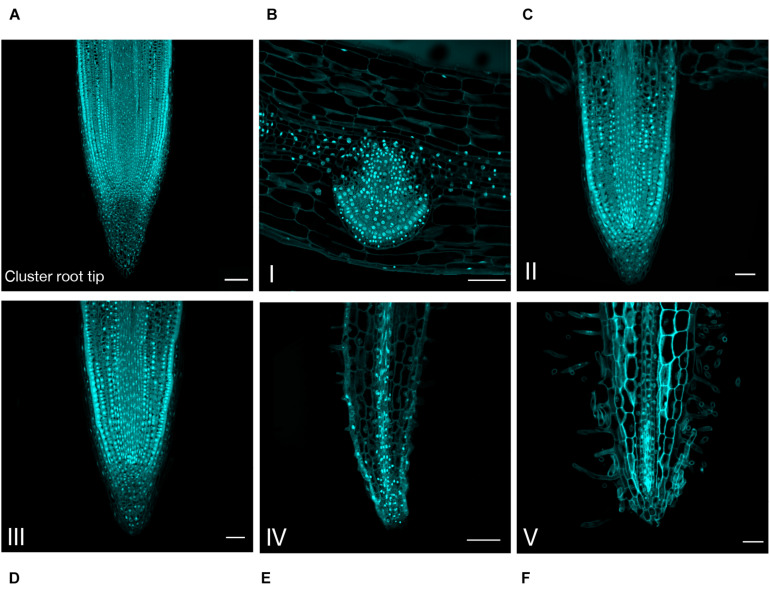
Anatomical study of the cluster root tip, and rootlet tips at different stages of development. DAPI was used to stain 8-μm thin longitudinal sections of **(A)** cluster root tip, and rootlet tips at **(B)** stage I, **(C)** stage II, **(D)** stage III, **(E)** stage IV, **(F)** stage V. Scale bars: **(A–F)** 50 μm.

**FIGURE 4:**
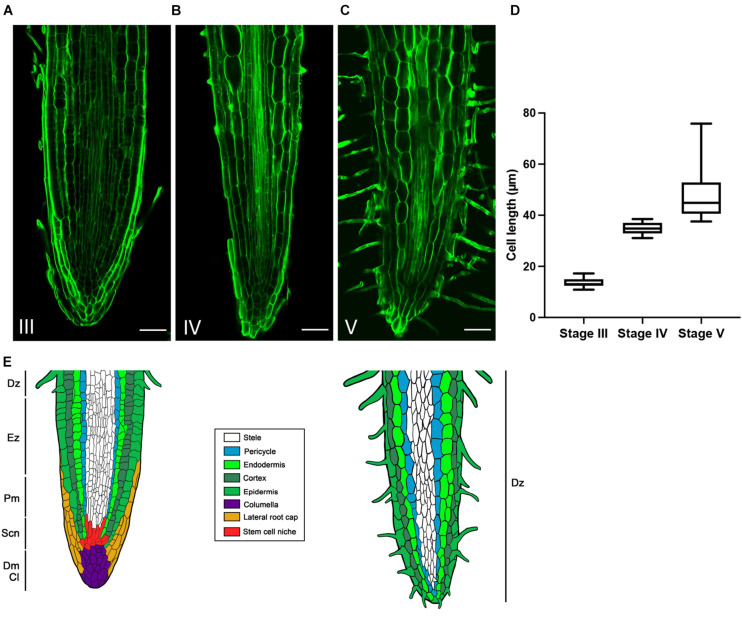
Evolution of cortical cells in the rootlet tips from stage III to V. Calcofluor was used to stain 100-μm thin longitudinal sections of rootlets. Typical images of rootlet tips at **(A)** stage III, **(B)** stage IV, and **(C)** stage V, are shown. Cell lengths were measured with Cell-o-Tape along 300 μm following a cortical cell line starting from the organization center **(D)** Cell lengths of a cortical line from stage III to V (*n* = 11). **(E)** Schematic representation of the evolution of rootlet tip before and after the developmental shift. Dz, differentiation zone; Ez, elongation zone; Pm, proximal meristem; Scn, stem cell niche; Dm, distal meristem; Cl, Columella. Scale bars: **(A–C)** 50 μm.

In order to confirm this hypothesis and to describe more accurately the developmental evolution at a tissular level, the rootlet meristems were observed at different stages of development with DAPI staining to monitor cell divisions (Figure 3). Up to stage III, rootlet tips showed a pattern similar to that of the cluster root tip (Figure 3A), with a typical root cap and a characteristic stem-cell niche, giving rise to cell lines with dividing activity (Figures 3B–D). Nonetheless, from stage IV to stage V, quite different patterns were observed including a decline followed by the complete disappearance of mitotic activities (Figures 3E,F). To support these findings, we performed confocal microscopy analyses on longitudinal sections stained with calcofluor-white dye in order to measure cell lengths of a cortical lineage from stage III to V (Figures 4A–C). Small meristematic cells with an average size of 13.7 μm were found at stage III, elongating cells with an average size of 35.1 μm at stage IV, and fully differentiated cells with an average cell size of 48.8 μm at stage V (Figure 4D). At this final stage of development, the vasculature has reached the apex of the rootlet, the root cap has disappeared and root hairs have covered the tip.

Finally, the loss of cell division activity, the elongation of the cells, the root hairs covering the apex and the central cylinder reaching the tip of the rootlet, reveal the exhaustion of the meristem and the complete differentiation of the rootlet, but nevertheless, it remains well-structured and organized without damage or swelling, as illustrated in the summary drawing (Figure 4E).

### Meristem Molecular Markers

**FIGURE 5:**
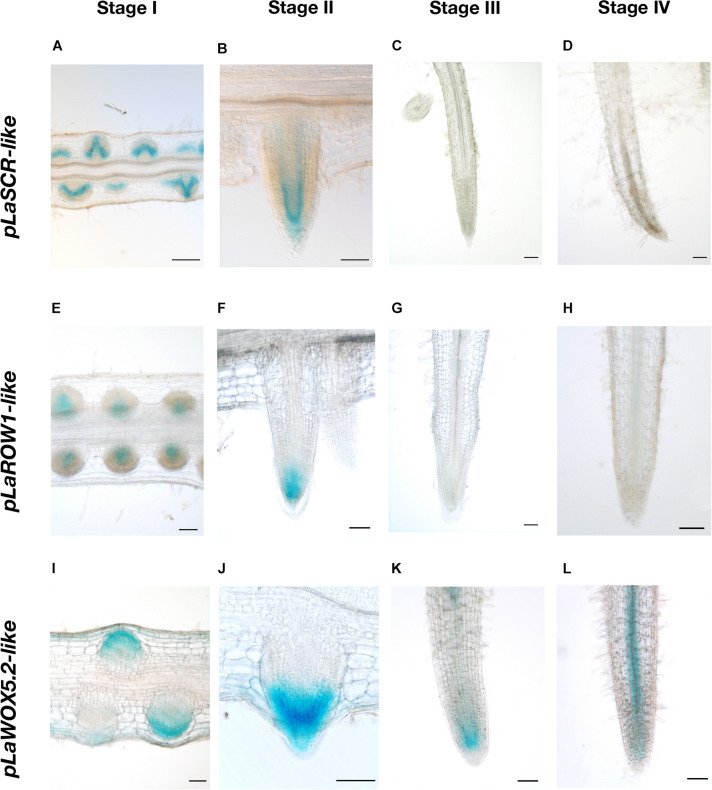
Tissular expression patterns of three meristematic markers during rootlet development. The promoter activities of three genes typically expressed in the meristematic zone were studied with a GUS-reporter-based analysis on 80-μm longitudinal sections of transgenic hairy-root tips. Images from stages I to IV are shown for each promoter **(A–D)** *pLaSCR-like:GUS*, **(E–H)** *pLaROW1-like:GUS*, **(I–L)** *pLaWOX5.2-like:GUS.* Scale bars: **(A–L)** 100 μm.

In order to characterize the development of the rootlet tip at a molecular level, we monitored the tissular expression of some canonical meristematic markers during the rootlet development. GUS reporter constructs, introduced in white lupin by hairy root transformation, were used to monitor the expression patterns of these genes. We observed GUS staining patterns in hairy-roots morphologically similar to young wild cluster roots and whose transformation was confirmed by the visualization of a constitutive DsRed marker. All the GUS reporter genes were highly expressed in the earliest rootlet primordia stages (Figure 5). In stage I (primordia) and II (post-emergence), the *pLaSCR-*like*:GUS* construct was expressed in the organizing center and endodermal cells (Figures 5A,B), and the *pLaROW1-like:GUS*, in the proximal zone above the organizing center (Figures 5E,F). These expression profiles are fully comparable to the canonical profiles observed in the meristem of the model plant *A. thaliana* (Sabatini et al., 2003; Zhang et al., 2015). p*LaWOX5.1-like:GUS* did not show a specific expression pattern (data not shown), whereas p*LaWOX5.2-like:GUS* expression was localized in the rootlet primordia, and we therefore chose this gene for further analysis. *pLaWOX5.2-like:GUS* was expressed in the apex of the rootlet at stages I and II but more broadly than expected by strict analogy with *A. thaliana* meristem (Pi et al., 2015) (Figures 5I,J). Nonetheless, the three meristematic molecular markers tested highlighted that a typical meristematic zone is present at the tip of the rootlet in the very first developmental stages. Then, from the growing stage III, GUS staining completely disappeared for the p*LaSCR-like:GUS* and *pLaROW1-like:GUS* constructs (Figures 5C,G), while it was still maintained for *pLaWOX5.2-like:GUS* (Figure 5K). At stage IV, we mainly observed GUS staining in the vascular tissues of hairy-roots expressing *pLaWOX5.2-like:GUS*, while the intensity of the staining in the meristematic zone weakened, confirming the very special expression profile of the *LaWOX5-like* genes (Figure 5L). For the other markers, there was no more staining in the root tissues from stage III onward (Figures 5D,H).

These results confirm the previous anatomical observations. Indeed, a canonical meristematic zone is present at the rootlet tip at the early stages of development, and gradually depletes during the growth phase until the determinate state.

### Spatial Transcriptomic Analysis of the Rootlet Life

**FIGURE 6:**
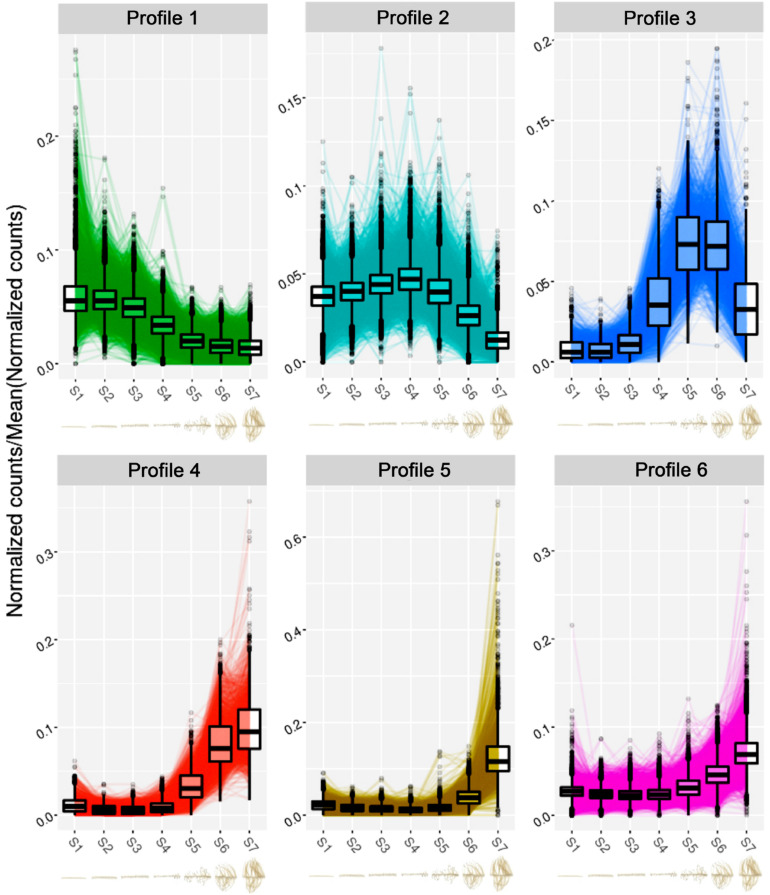
Clustering analysis of the normalized expression profiles of the differentially expressed transcripts between S1 and S7 sections of the RNA-seq experiment. Normalized gene expression profiles come from our previous RNA-seq study (Hufnagel et al., 2020). The clustering was performed with the Coseq package in DIANE (Cassan et al., 2021). The numbers of genes clustered in each gene expression profile are the following: Profile 1: 1,792; Profile 2: 2,129; Profile 3: 411; Profile 4: 482; Profile 5: 584; Profile 6: 1,836. Below each profile, the cluster root sections from S1 to S7 of the RNA-seq analysis are presented.

In order to get a global vision of gene expression during the different phases of the rootlet development, from the pre-emergence to the final growth stage, the transcriptome of each of the 8 zones of one cluster of rootlets (named S0 to S7), previously carried out (Hufnagel et al., 2020) was analyzed using DIANE, a tool for the analysis of high throughput gene expression data (Cassan et al., 2021). A total of 7,234 differentially expressed transcripts (DETs), including 3,329 up-regulated and 3,905 down-regulated genes, were detected comparing the pre-emergence stage (S1) and final growing stage (S7). The time courses of expression of these DETs along the cluster of rootlets were then analyzed and grouped into 6 spatial/temporal profiles (Figure 6). Interestingly, the profiles present maxima of transcript accumulation in, respectively, the S1/S2 (profile 1), S4 (profile 2), S5/S6 (profile 3) and S6/S7 (profile 4) and S7 (profile 5 and 6), revealing five successive waves of developmental stages. To gain an insight into the differences in biological processes that are likely to characterize these successive waves of gene expression, a GO enrichment analysis was performed on the set of DETs from the six profiles (Supplementary Figure 2 and Supplementary Table 1). Among the most significantly overrepresented GO terms for the profile 1, we find several GO terms associated with “auxin-activated signaling pathway,” “regulation of growth meristem,” and “procambium histogenesis,” reflecting the biological processes of root meristem organogenesis, with for instance, the presence in this profile of genes encoding *PLETHORA-like* (*Lalb_Chr01g0003341, Lalb_Chr01g0003331*), *PUCHI-like* (*Lalb_Chr07g0177601*, *Lalb_Chr13g0303751*, *Lalb_Chr15g0086951*, *Lalb_Chr18g0055601*), *LBD16-like* (*Lalb_Chr02g0142301*, *Lalb_Chr06g0162491*) and *WOX5-like* (*Lalb_Chr04g0250731*, *Lalb_Chr18g0052701).* All these genes are known to be involved in morphogenesis of the early lateral root primordium. In the profile 2, a large proportion of DETs are enriched into the category of biological processes as “cell division” and “microtubule-based movement” including genes such as *ANTEGUMENTA-like* (*Lalb_Chr16g0381611*), *BABYBOOM/PTL4-like* (*Lalb_Chr06g0168521*), *ROW1-like* (*Lalb_Chr01g0019561*), and *PLETHORA1/2-like* (*Lalb_Chr19 g0138171*) and a large number of cyclins (*Lalb_Chr10g0101501*, *Lalb_Chr20g0108171*, *Lalb_Chr10g0106101*, *Lalb_Chr09g03212 81*, *Lalb_Chr08g0241201*, *Lalb_Chr11g0063951*, *Lalb_Chr22g 0354811* and others). All these genes are expressed in meristematic and division-competent states. As expected, profiles 1 and 2 are therefore clearly characteristic of meristem formation and activity.

In profiles 3 and 4, a clear shift occurs. Seventeen genes encoding Pectin Methyl Esterase Inhibitors (PMEIs) are found in profile 3. Pectin Methyl Esterase Inhibitors can be considered as a determinant of cell adhesion, cell wall porosity and elasticity, as well as a source of signaling molecules released upon cell wall remodeling. In profile 4, a strong enrichment for “cell wall organization” and “oxidative stress” marker genes including peroxidase gene family, stand out. Deep changes in cell walls seem to characterize these stages of rootlet development.

Both profiles 5 and 6 show a peak of transcript accumulation in section S7. In profile 5, physiological responses to Pi starvation appear with the biological functions “cellular responses to phosphate starvation” and “dephosphorylation” including the phosphatase acid gene family. This is an expected response as the plants were grown in Pi deficient condition. In profile 6, the enriched GO terms detected the term “tricarboxylic acid cycle” (Supplementary Table 1) in which are found genes coding for enzymes such as citrate synthase (*Lalb_Chr04g0248661*, *Lalb_Chr25g 0283641*, *Lalb_Chr10g0106821*), malate dehydrogenase (*Lalb_Chr21g0312041*, *Lalb_Chr13g0303211*), isocitrate dehydrogenase (*Lalb_Chr10g0104131*), succinate dehydrogenase (*Lalb_Chr23g0273091, Lalb_Chr16g0377871*) and also three phosphoenolpyruvate carboxylase (*Lalb_Chr01g0006821*, *Lalb_Chr25g0285901*, *Lalb_Chr20g0109301*). These root PEPcase are typical of lupin root metabolism. In the late stages of sampling (S6-S7), rootlets reached their maximum of physiological activity with activation of PEPcases and TCA cycle supporting the burst of citrate exudation characteristic of the response of white lupin to phosphate deficiency. Interestingly, among the transcripts upregulated at the late developmental stages, a huge over-representation of transcripts involved in transmembrane transport occurs (Supplementary Figure 2) with 51 genes encoding for members of the plasma membrane transporters involved in phosphate, nitrate, ammonium, potassium, sulfate, iron, zinc, and calcium transport (Table 1). Other genes implicated in H^+^, sugar or water transport, ABC transporters and major facilitator superfamily are likewise present. Many homologs of membrane transport genes involved in nutrition are found to be highly expressed in later phases of rootlet development. These data confirm the total integrity of the plasma membrane and its high activity level at these stages.

**TABLE 1:** List of plasma membrane transport genes overexpressed in the late stages of rootlet development.

Lupin gene code	Arabidopsis gene code	Gene description	Element	Profile number
Lalb_Chr20g0113031	AT2G37330	Aluminum sensitive 3 (ALS3)	Aluminum	Profile 5
Lalb_Chr07g0185661	AT4G21120	Amino acid transporter 1 (AAT1)	Amino acid	Profile 6
Lalb_Chr14g0363171	AT5G40780	Lysine histidine transporter 1 (LHT1)	Amino acid	Profile 6
Lalb_Chr03g0042381	AT3G09330	Amino acid transporter (AVT1G)	Amino acid	Profile 6
Lalb_Chr13g0295001	AT3G13620	Polyamine uptake transporter 4 (PUT4)	Amino acid	Profile 6
Lalb_Chr17g0344301	AT3G13620	Polyamine uptake transporter 4 (PUT4)	Amino acid	Profile 6
Lalb_Chr07g0184111	AT2G38290	Ammonium transporter 2 (AMT2)	Ammonium	Profile 4
Lalb_Chr18g0052441	AT1G80660	H(+)-ATPase 9	ATPase	Profile 4
Lalb_Chr20g0116471	AT1G80660	H(+)-ATPase 9	ATPase	Profile 5
Lalb_Chr13g0303081	AT5G61350	Protein kinase superfamily protein (CAP1)	Calcium	Profile 5
Lalb_Chr14g0362681	AT4G22120	Calcium-permeable stretch activated cation channel (CSC1)	Calcium	Profile 4
Lalb_Chr05g0218691	AT2G29120	Glutamate receptor 2.7 (GLR2.7)	Calcium	Profile 5
Lalb_Chr05g0218701	AT2G29120	Glutamate receptor 2.7 (GLR2.7)	Calcium	Profile 5
Lalb_Chr25g0283091	AT3G08040	Ferric reductase defective (FRD3)	Citrate	Profile 5
Lalb_Chr13g0291391	AT2G38460	Iron-regulated transporter 1 (IRT1)	Iron	Profile 5
Lalb_Chr14g0367651	AT1G60960	Iron regulated transporter 3 (IRT3)	Iron	Profile 5
Lalb_Chr24g0394061	AT5G59520	ZRT/IRT-like protein 2 (ZIP2)	Iron	Profile 5
Lalb_Chr02g0149651	AT4G00910	Aluminum-activated malate transporter 10 (ALMT10)	Malate	Profile 5
Lalb_Chr03g0027601	AT1G08100	High-affinity nitrate transporter 2.2 (NRT2.2)	Nitrate	Profile 5
Lalb_Chr05g0228311	AT1G12940	Nitrate transporter2.5 (NRT 2.5)	Nitrate	Profile 4
Lalb_Chr07g0183011	AT5G50200	High-affinity nitrate transporter (NRT3.1)	Nitrate	Profile 6
Lalb_Chr08g0234171	AT2G23980	Cyclic Nucleotide-Gated Channel 6 (CNGC6)	Nitrate	Profile 4
Lalb_Chr01g0000701	AT3G47420	Phosphate starvation-induced glycerol-3-phosphate permease (G3PP1)	Phosphate	Profile 6
Lalb_Chr04g0259111	AT2G32830	Phosphate transporter 1;5 (PHT1;5)	Phosphate	Profile 4
Lalb_Chr05g0219741	AT1G76430	Phosphate transporter 1;9 (PHT1;9)	Phosphate	Profile 4
Lalb_Chr09g0328731	AT1G76430	Phosphate transporter 1;9 (PHT1;9)	Phosphate	Profile 5
Lalb_Chr10g0094631	AT2G32830	Phosphate transporter 1;5 (PHT1;5)	Phosphate	Profile 5
Lalb_Chr04g0259111	AT2G32830	Phosphate transporter 1;5 (PHT1;5)	Phosphate	Profile 4
Lalb_Chr09g0330281	AT2G40540	Potassium transporter 2 (POT2)	Potassium	Profile 6
Lalb_Chr24g0393761	AT4G10310	High-affinity K + transporter 1 (HKT1)	Potassium	Profile 5
Lalb_Chr09g0332731	AT4G23700	Cation/H + exchanger 17 (CHX17)	Sodium	Profile 5
Lalb_Chr05g0210921	AT5G26340	Sugar transport protein 13 (STP13)	Sugar	Profile 6
Lalb_Chr06g0160591	AT5G26340	Sugar transport protein 13 (STP13)	Sugar	Profile 5
Lalb_Chr20g0123111	AT1G22150	Sulfate transporter 1;3 (Sultr1;3)	Sulfate	Profile 4
Lalb_Chr01g0018841	AT2G16850	Plasma membrane intrinsic protein 2;8 (PIP2;8)	Water	Profile 5
Lalb_Chr02g0142171	AT2G37170	Plasma membrane intrinsic protein 2;2 (PIP2;2)	Water	Profile 5
Lalb_Chr02g0142181	AT5G60660	Plasma membrane intrinsic protein 2;4 (PIP2;4)	Water	Profile 5
Lalb_Chr03g0027361	AT5G60660	Plasma membrane intrinsic protein 2;4 (PIP2;4)	Water	Profile 6
Lalb_Chr24g0401831	AT1G05300	Zinc transporter 5 (ZIP5)	Zinc	Profile 5
Lalb_Chr02g0151441	AT2G36380	Pleiotropic drug resistance 6 (ABCG34)	Others	Profile 4
Lalb_Chr03g0024501	AT3G21090	ABC-2 type transporter family protein (ABCG15)	Others	Profile 5
Lalb_Chr04g0261571	AT1G01340	Cyclic Nucleotide Gated Channel 10 (CNGC10)	Others	Profile 5
Lalb_Chr08g0242031	AT1G34580	Major facilitator superfamily protein	Others	Profile 4
Lalb_Chr08g0242041	AT1G34580	Major facilitator superfamily protein	Others	Profile 5
Lalb_Chr16g0391341	AT4G10770	Oligopeptide transporter 7 (OPT7)	Others	Profile 6
Lalb_Chr19g0130401	AT4G27970	SLAC1 homolog 2 (SLAH2)	Others	Profile 4
Lalb_Chr20g0113531	AT1G15520	ATP-binding cassette G40 (ABCG40)	Others	Profile 6
Lalb_Chr21g0305601	AT1G80760	NOD26-like intrinsic protein 6;1 (NIP6;1)	Others	Profile 4
Lalb_Chr24g0402281	AT1G15520	ATP-binding cassette G40 (ABCG40)	Others	Profile 4
Lalb_Chr25g0284471	AT1G16310	Cation efflux family protein	Others	Profile 5

*The plasma membrane transport genes from profiles 4, 5, and 6 of the clustering analysis are listed.*

In order to validate the results from RNA-seq analysis, transcripts of 6 selected genes, which were significantly expressed and mostly implied in nutrient transport were analyzed by Real-Time-qPCR (Supplementary Figure 3). Genes considered for this analysis are coding for a Pi transporter (*LaPHT1;5-like Lalb_Chr04g0259111* – profile 4), a sulfate transporter (*LaSULTR1;3-like Lalb_Chr20g0123111* – profile 4), a H(+)-ATPase (*LaHA9-like Lalb_Chr18g0052441* – profile 4), an iron transporter (*LaIRT1-like Lalb_Chr13g0291391* – profile 5), a nitrate transporter (*LaWR3-like Lalb_Chr07g0183011* – profile 6) and an ammonium transporter (*LaNH4-like Lalb_Chr07g0184111* – profile 4). The expression of these 6 genes was assessed in stage II to V of rootlet development, in the cluster root tip, and in the link section corresponding to the attachment of the cluster root to the primary root where no rootlets are developed. The results obtained were fully consistent with the RNA-seq analysis (Supplementary Figure 3).

### Rootlet Functional Phase

As we could observe in the late stages of rootlet development a burst of genes implied in responses to phosphate starvation as well as genes implied in phosphate transport, we decided to assess phosphate absorption and organic acid exudation during the course of rootlet development as defined in Figure 1 in order to detect at which stage of development the Pi metabolism is boosted.

**FIGURE 7:**
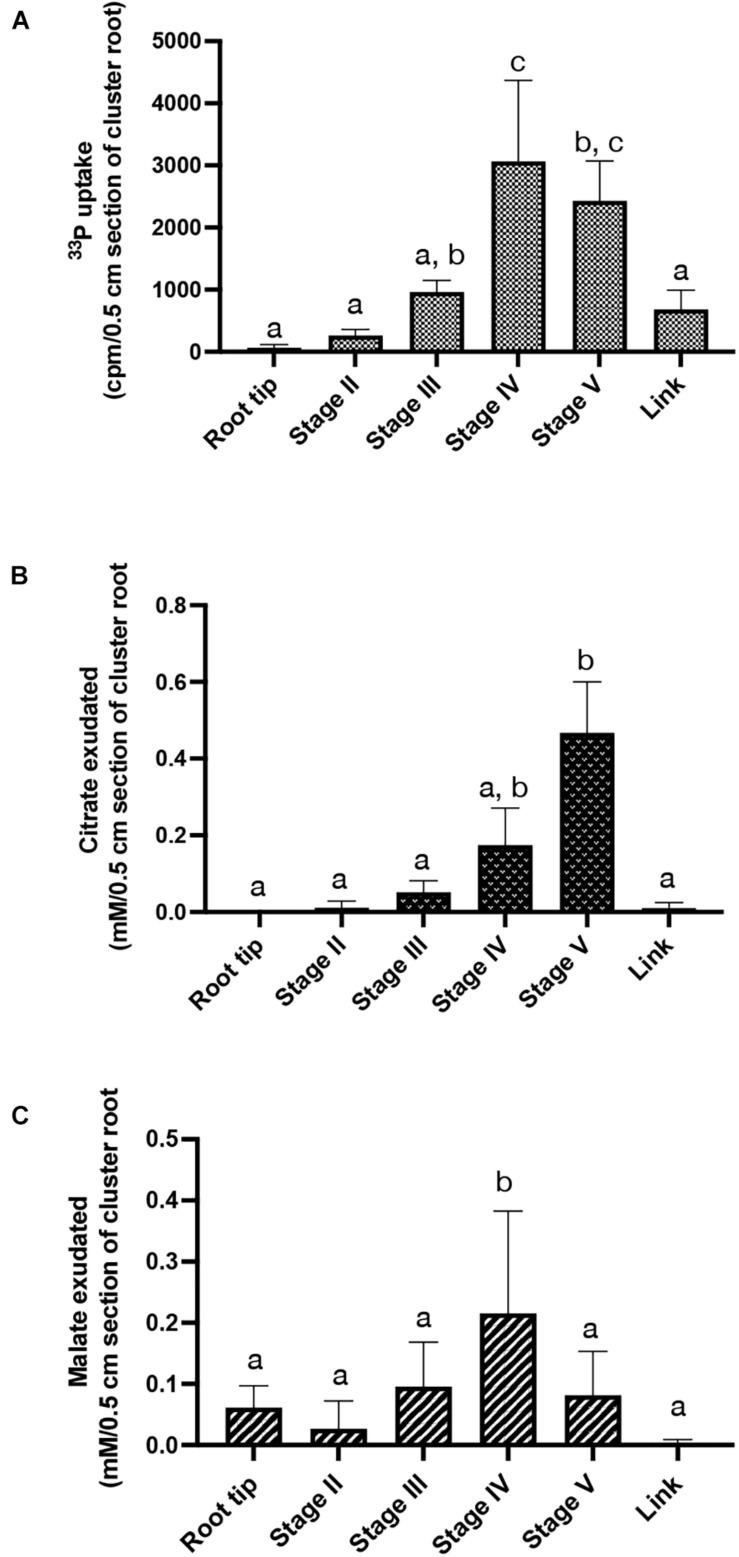
Physiological activities along rootlet development. **(A)** ^33^P influx in 0.5 cm cluster root sections, root tip and link segment. Values are means ± SD of three biological replicates coming each from 6 cluster roots sampled across 6 white lupin plants (*n* = 3). **(B)** Citrate and **(C)** malate exudations quantified in 0.5 cm cluster root sections, root tip and link segment. Values are means ± SD of five biological replicates coming each from 6 cluster roots sampled across 6 white lupin plants (*n* = 5).

Uptake of Pi was investigated by performing ^33^P labeling assays using excised cluster root segments corresponding to stage II to V of rootlet development. Pi influx significantly increased in stages IV and V, with at least a 2-fold higher uptake compared to stages II and III (Figure 7A).

Citrate and malate exudations were also quantified in stage II to V of rootlet development, on the cluster root tip and the link segment (Figures 7B,C). Malate exudation remained constant in the root tip and the stages II, III and V but a two-fold increase was observed in stage IV (Figure 7B). No detectable excretion of citrate was observed in the cluster root tip. However, a gradual increase in citrate exudates was detected during the rootlet development. The amount of released citrate was at least 100% to 150% higher in stage IV and stage V, respectively, compared to the cluster root tip, link and stages II and III (Figure 7C). These data indicate that rootlets exudate large amounts of citrate as soon as they reach their final length. The same profile was obtained with a normalization by fresh weight (data not shown).

Taken together, these findings indicate that rootlets at the end of the growing phase evolve in specialized structures able to uptake large amounts of Pi and to exudate large amounts of organic acids.

## Discussion

### From the End of Its Growth, the Rootlet Is a Highly Active Nutrition-Specialized Organ

The formation of clusters of rootlets constitute a spectacular developmental and functional adaptation of white lupin to cope with phosphorus deficiency (Neumann and Martinoia, 2002). Their physiological activity has been widely documented (Dinkelaker et al., 1989; Neumann et al., 2000; Massonneau et al., 2001; Tomasi et al., 2009; Müller et al., 2015). However, few studies have been dedicated to the development of these structures. The very early developmental stages, including the initiation and the formation of rootlet primordia, have been described (Cheng et al., 2011; Gallardo et al., 2019). They appeared to be very similar to the lateral root formation in the model plant *A. thaliana*, with the establishment of an auxin maximum and the progressive organogenesis of a new meristem from the inner tissues of the root. The development of the rootlet itself is then more specific. Many works studied long cluster roots bearing several clusters of rootlets of different age: emergent, juvenile, mature and senescent, but did not describe the dynamics of rootlet development within a single cluster (Neumann and Römheld, 1999; Weisskopf et al., 2006; Tomasi et al., 2008; Wang et al., 2010; Tiziani et al., 2020). We focused on young white lupin root system, with cluster roots bearing a single cluster and studied its rootlets from emergence to final growth. It is well known that rootlet growth is determinate (Skene, 1998; Neumann and Römheld, 1999; Watt and Evans, 1999). However, how rootlets tips shift from a growing to a determinate state is not yet clear. Indeterminate root growth is sustained by the root apical meristem (RAM). The maintenance of the stem cell niche, composed by the mitotically less active quiescent center and the initial cells, orchestrates a proper balance between cell division and cell differentiation (Van Den Berg et al., 1997). The regulation of the meristematic zone implies complex regulatory mechanisms involving phytohormones, transcription factors such as WOX5 and SCR, and regulatory proteins like ROW1 (Drisch and Stahl, 2015; Zhang et al., 2015). We therefore identified *LaWOX5-like*, *LaSCR-like* and *LaROW1-like* as white lupin homologs of these main regulators of the stem cell niche function. At the beginning of rootlet development, anatomical observations and RNA-seq analysis demonstrated the establishment and maintenance of a normal root meristem with high dividing activities along with elongation and differentiation zones. However, the anatomical description of the rootlet development highlighted a major transition between emergence and rootlet growth arrest at stage IV, corresponding to the S5 and S6 portions of the RNA-seq sampling. Our results suggest an exhaustion of stem cells and a complete differentiation of the rootlet which becomes determinate. It is known that root determinacy is usually related to developmental changes within the root apical meristem and that any disruption in the balance between cell division and cell differentiation would lead to the deregulation or even the exhaustion of the meristem. These changes are often accompanied with multiple growth defects including swelling or program cell death (Shishkova et al., 2008; Hernández-Barrera et al., 2011). However, no tissue damages have been observed in the rootlets studied. On the contrary, our RNA-seq analysis showed an impressive burst of metabolic and membrane transport activities in the S6/S7 samples. In the time course of the analysis, the final determinate stage is clearly reached but the rootlet is not yet senescent and its physiological activities are high. Interestingly, RNA-seq data analysis suggested an increase in membrane proteins dedicated to the uptake of many essential nutrients such as phosphate, nitrate, ammonium, potassium, sulfate, iron, zinc or calcium. Such a result was already present in previous white lupin transcriptome analysis (Secco et al., 2014; Wang et al., 2014; Zanin et al., 2019), but the focus was only on Pi nutrition. At the end of the growing phase, massive secretion of protons and citrate into the rhizosphere may affect not only the availability of phosphate, but also the solubility of other nutrients. The simultaneous increase of several membrane transporters boosts overall nutrient-uptake efficiency. Finally, our study underlines that the rootlet becomes a hyper-specialized organ dedicated to nutrient acquisition just after its growth arrest. Its high physiological activities at its determinate state lead to the complete nutrient exploitation of a small patch of soil.

### White Lupin Rootlet Determinacy Is Different From *A. thaliana* Primary Root Growth Arrest in Response to Pi Deficiency

The primary root of *A. thaliana* has been extensively studied for its growth arrest in response to Pi starvation (Sánchez-Calderón et al., 2005; Svistoonoff et al., 2007; Péret et al., 2014). However, the QTL analysis based on the differential responses of *Col* and *Ler A. thaliana* accessions to Pi deprivation identified that the main determinants of this process reside in an alteration in the functioning of primary root apex (Reymond et al., 2006) while our results show that it is not the case for white lupin rootlets. Indeed, when the tip of the *A. thaliana* primary root detects external Pi limitation, a rapid inhibition of cell elongation occurs in the transition zone followed by a progressive arrest of cell proliferation (Svistoonoff et al., 2007; Ward et al., 2008; Ticconi et al., 2009; Müller et al., 2015). The fine molecular dissection of this process has highlighted the involvement of LPR1-PDR2 and STOP1-ALMT modules leading to ROS regeneration and callose deposition in the plasmodesmata of the root apical meristem, inducing the root tip decay (Balzergue et al., 2017; Mora-Macías et al., 2017; Gutiérrez-Alanís et al., 2018). In opposition to the *A. thaliana* primary root response, the anatomic and molecular descriptions of white lupin rootlet development show a rapid exhaustion of stem cell niche after emergence, and then, an arrest of cell divisions while elongation goes on, along with the differentiation of all rootlet cells. As quoted earlier, the necrotic appearance described in *A. thaliana* primary root (Sánchez-Calderón et al., 2005) was not observed in the rootlets studied. In opposition, rootlets remain fully active with a burst of membrane transporters, phosphatases, PEP-carboxylase and pentose phosphate cycle activities, revealing a very intense metabolic phase. All these results led to propose that the determinate growth of the white lupin rootlet is not similar to that of *A. thaliana* primary root, but rather looks like a transition from canonical meristematic structures to differentiated tissues highly active and specialized in nutritional activities.

### Root Determinacy Is a Hallmark of Plants Growing in Adverse Conditions to Cope With Water or Pi Deficiency

In many plants, the determinacy of root growth is only induced by mechanical or nutritional stresses (Shishkova et al., 2008). In white lupin, or some Cactaceae, a more regular determinacy appears in the root system (Dubrovsky, 1997; Shishkova et al., 2008). These plants naturally grow in adverse environments. In white lupin, formation of cluster roots is a response to Pi shortage and allows the exploitation of a low mobile Pi pool. We showed that rootlet reaches its higher metabolic activity when it stops growing. In the same way, recent transcriptome analysis has underlined the role of water stress in the regulation of the cactus *Pachycereus pringlei* primary root determinacy to favor the development of the upper part of the root system (Rodrìguez-Alonso et al., 2018). In maize (Varney and Mccully, 1991) and, more recently, in other cereals such as rice (Rebouillat et al., 2009) or pearl millet (Passot et al., 2018), lateral roots with a determinate fate have also been described. The proportion of short and long lateral roots was shown to be under the control of both environmental and genetic factors (Vejchasarn et al., 2016). In maize, the presence of short determinate roots is genetically controlled and varies between cultivars (Dowd et al., 2019). Short determinate maize lateral roots have open xylem vessels down to the tip, favoring high water conductivity (Wang et al., 1994). However, water deficit conditions delay the determinacy program to give priority to root elongation and water foraging (Dowd et al., 2020). It is interesting to note that root determinacy in cactus and white lupin is an adaptation to low mobile resources, rain water and Pi pools, respectively, whereas it is delayed in cereals to favor a deeper root system, illustrating the root dilemma between maximizing soil exploitation or soil exploration.

The fine study of white lupin rootlet development gave a clear illustration of this dilemma showing that exhaustion of the meristematic zone is required to build a short organ specialized in soil mining to optimize solubilization and absorption. The intricate molecular controls of the determinate growth leading to a complete differentiation into specialized roots dedicated to mining instead of foraging, is fully unknown and the study of white lupin rootlet development provides a powerful model to gain insight into this question.

## Data Availability Statement

The original contributions presented in the study are included in the article/Supplementary Material, further inquiries can be directed to the corresponding authors.

## Author Contributions

PD, LM, and TL contributed to conception and design of the study. TL performed the main experiments. CC and LB provided technical assistance in molecular biology and hairy root transformation. LM supervised microscope experiments. AS and PD analyzed the transcriptome data. BH, BP, and FD generated tools and analyzed data. LM and TL wrote the article. All authors contributed to manuscript revision, read, and approved the submitted version.

## Conflict of Interest

The authors declare that the research was conducted in the absence of any commercial or financial relationships that could be construed as a potential conflict of interest.

## Publisher’s Note

All claims expressed in this article are solely those of the authors and do not necessarily represent those of their affiliated organizations, or those of the publisher, the editors and the reviewers. Any product that may be evaluated in this article, or claim that may be made by its manufacturer, is not guaranteed or endorsed by the publisher.

## Footnotes

1
www.whitelupin.fr

2
http://primer3plus.com
