# Supplementary material for: Dynamic Development of White Lupin Rootlets Along a Cluster Root
Source: Front Plant Sci. 2021 Sep 7;12:738172. doi: 10.3389/fpls.2021.738172 (PMC8452988; doi:10.3389/fpls.2021.738172)
Supplement: Supplementary file 1 [file Data_Sheet_1.pdf]

Supplementary Material

1 Supplementary Data

2 Supplementary Figures and Tables

2.1 Supplementary Figures 1

Figure S1: Phylogenetic analysis and RNA-seq expression data of genes used as meristematic markers. Trees were constructed using the FastTree workflow of NGphylogeny (htttp://www.ngphylogeny.fr). Branches lengths are displayed. Homologs selected for further analysis are highlighted. For each white lupin homologues, the RNA-seq expression data along a cluster root are shown.

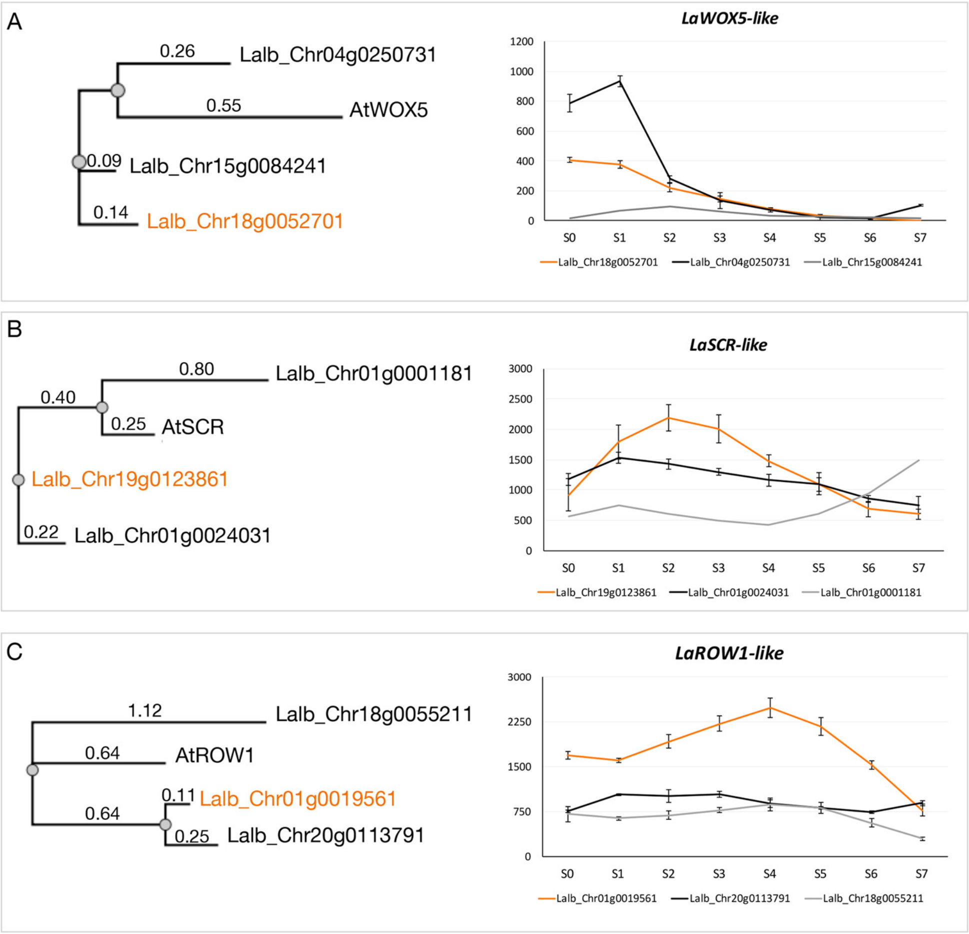

2.2 Supplementary Figures 2

Figure S2: Gene ontology enrichment analysis in the six DETs profiles from the clustering data. Biological process GO terms (under 0.05 in Fisher's exact tests) were used for the GO analysis, the nodes are color-coded by their adjusted p-value, and shifted in the x-axis depending on the number of genes matching this ontology.

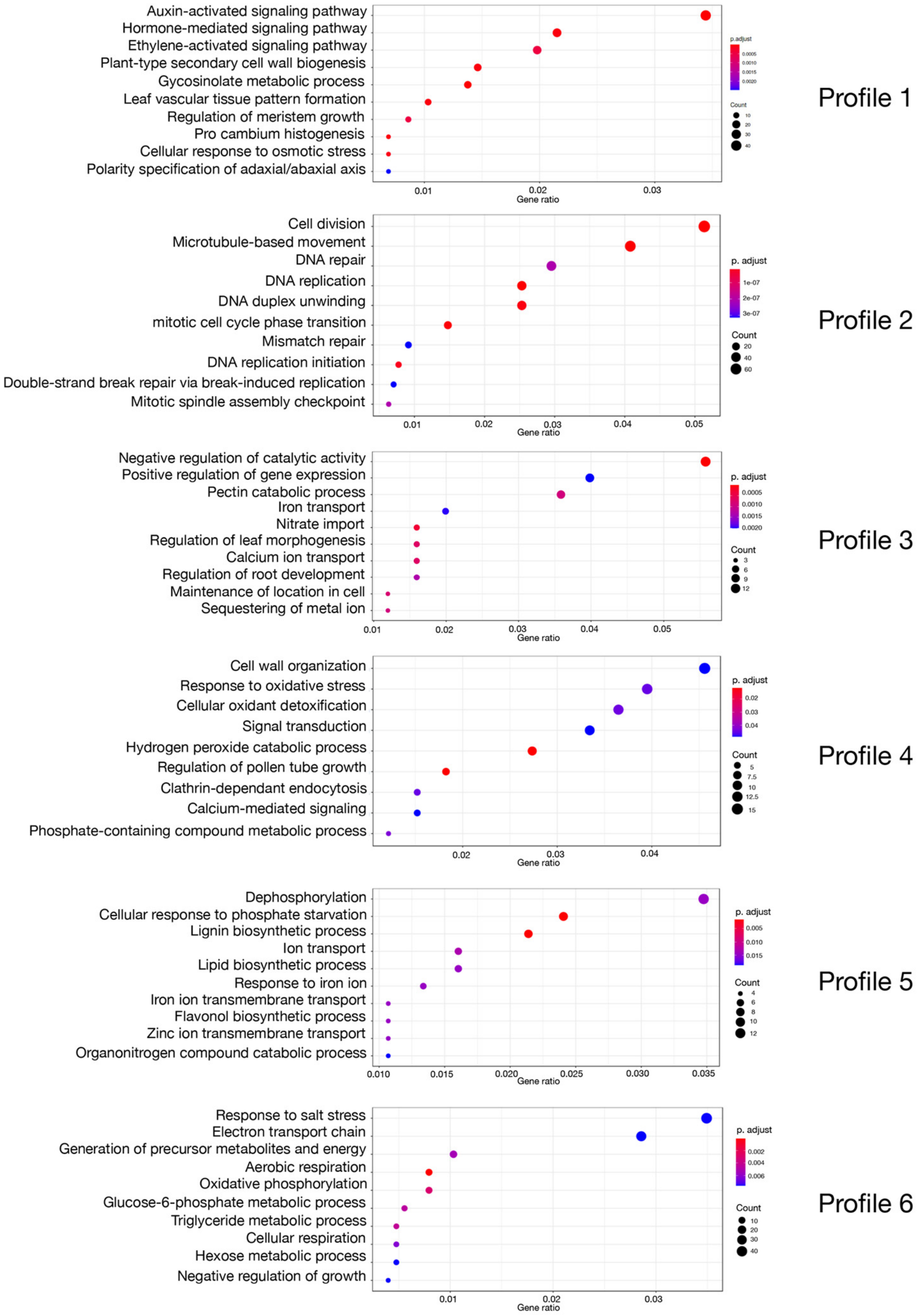

2.3 Supplementary Figures 3

Figure S3: RT-qPCR expression patterns of some genes upregulated in the S7 section of the RNA-seq analysis and involved in nutrient transport. (A) *LaPHT1;5-like* (*Lalb_Chr04g0259111*) Pi transporter, (B) *LaSUTR1;3-like* (*Lalb_Chr20g0123111*) sulfate transporter, (C) *LaHA9-like* (*Lalb_Chr18g0052441*) H(+)-ATPase, (D) *LaIRT1-like* (*Lalb_Chr13g0291391*) iron transporter, (E) *LaWR3-like* (*Lalb_Chr07g0183011*) nitrate transporter and (F) *LaNH4-like* (*Lalb_Chr07g0184111*) ammonium transporter. The expression level is relative to the reference gene *LaNORM1* (*Lalb_Chr07g0195211*). The values are means +/- SD of 5 biological replicates.

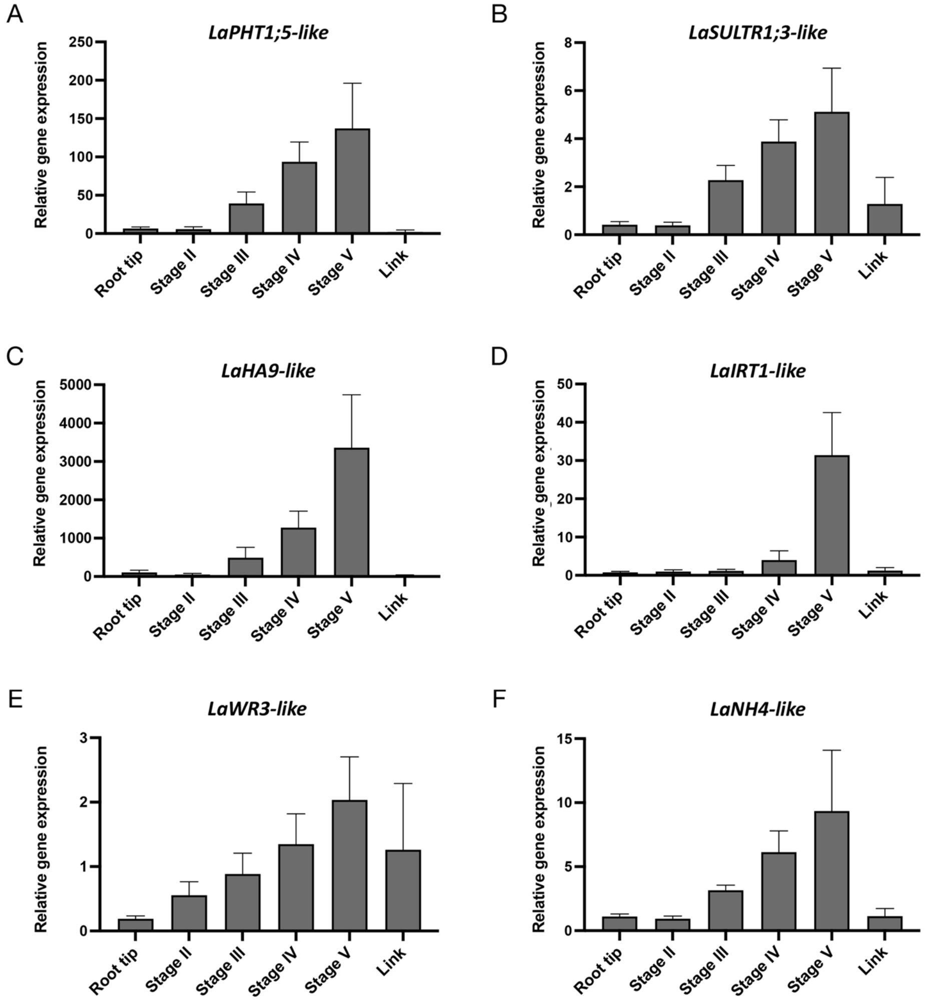

Table S1: Lists of DETs and GO enrichments for the 6 gene expression profiles from the clustering analysis. From RNA-seq data (Hufnagel et al, 2020), a clustering analysis of the normalized expression profiles of the 7,234 DETs between S1 and S7, and a GO enrichment analysis were performed with DIANE (Cassan et al., 2021). Six gene expression profiles were obtained and the results for each profile are given in the different sheets of the Excel file.

Table S2: Primer sequences used for the RT-qPCR analysis. The primer pairs were designed with Primer3plus program (http://primer3plus.com).
